# Contribution of dynamic contrast enhancement and diffusion-weighted magnetic resonance imaging to the diagnosis of malignant cervical lymph nodes

**DOI:** 10.1590/0100-3984.2018.51.3e3

**Published:** 2018

**Authors:** Angela M. Borri Wolosker

**Affiliations:** 1 MD, PhD, Attending Physician in the Head and Neck Sector of the Department of Diagnostic Imaging at the Escola Paulista de Medicina da Universidade Federal de São Paulo (EPM-Unifesp), Senior Physician for Grupo Fleury, São Paulo, SP, Brazil. E-mail: ambwolosker@yahoo.com.br.

The importance of imaging diagnosis in identifying and characterizing cervical lymph nodes is undeniable, especially in tumor staging, in which it influences decisions related to the initiation, adjustment, or discontinuation of treatment^(^^1^^)^. The limitations of conventional magnetic resonance imaging (MRI) for the differentiation between benign and malignant involvement are also recognized, because a reactive lymph node can be enlarged in the same way as a metastatic lymph node, and a normal-sized lymph node can be malignant^(^^2^^)^. Functional MRI techniques are increasingly used to aid in that differentiation.

Diffusion-weighted MRI sequences can identify change in cytoarchitecture and cell density, allowing the use of apparent diffusion coefficient (ADC) values for the characterization of small (4-9 mm) metastatic lymph nodes^(^^3^^)^, for which the morphology and size criteria would yield false-negative results^(^^4^^)^. In addition, many studies have demonstrated the importance of post-treatment assessment with diffusion-weighted imaging, suggesting that, by as soon as two weeks after the initiation of treatment, ADC values can indicate whether or not the prescribed pharmacological treatment has been successful^(^^5^^)^. The various techniques of dynamic study after injection of contrast medium allow characterization of vascular permeability and identification of neovascularization, suggestive of metastatic lymph nodes^(^^6^^)^. Spectroscopy demonstrates a change in the concentration of metabolites, which can guide the diagnosis^(^^3^^)^.

In the previous issue of Radiologia Brasileira, Cintra et al.^(^^7^^)^ analyzed 33 functional MRI studies of cervical lymph nodes, with the objective of identifying signs indicative of malignancy or benignity. The authors used diffusion-weighted imaging and dynamic contrast enhancement with perfusion/vascularity evaluation to characterize the lymph nodes, comparing those results with the postoperative pathology and fine needle aspiration biopsy findings.

The results showed that the diffusion-weighted imaging did not show statistically significant differences between benign and malignant lymph nodes. Dynamic contrast enhancement showed statistically significant results for two parameters: peak enhancement and relative enhancement. Malignant lymph nodes showed lower values of peak enhancement and higher values of relative enhancement. 

The authors commented on some discrepant results in the literature^(^^8^^)^, offering a critical analysis emphasizing that functional MRI has the potential to differentiate between malignant and benign lymph nodes if evaluated in association with the analysis of conventional images. They noted the limitations to functional MRI, such as the presence of artifacts (respiratory and involuntary swallowing movements) and areas of necrosis (which must be recognized and correctly interpreted), as well as the wide range of parameters and cut-off values used in the literature, which limit reproducibility and preclude comparisons across studies^(^^9^^)^.
